# Author's Reply

**DOI:** 10.1371/journal.pmed.0020405

**Published:** 2005-11-29

**Authors:** Amir Attaran

**Affiliations:** **1**University of OttawaOttawa, OntarioCanada

I am grateful for the reply of Jeff Sachs, John McArthur, and Guido Schmidt-Traub to my article [1,2]. Their reply, written on behalf of the United Nations (UN) Millennium Project, shows that even the leading thinkers of that organization possess beliefs about the Millennium Development Goals (MDGs) that contradict the factual evidence.

On one issue, the UN Millennium Project team agrees with me: progress on the MDGs is sometimes not well measured, such that it is impossible to know if the goals are on track to being fulfilled by their 2015 deadline.

But even on this issue, we disagree on the extent of the problem. The UN Millennium Project team writes that, because my analysis was limited to only the public health MDGs, I based my conclusions on only “the toughest measurement challenges,” and “generalize[d] incorrectly across the MDGs” [1]. In their view, if one leaves the difficult health MDGs aside, then “several other MDG indicators can be measured quite well” [1].

To determine whether this assertion is correct, I accessed the UN's own “Data Availability Analysis” [3] for 2005, which takes into account every one of the 48 UN-designated MDG indicators (really 65 indicators in 48 categories). For each indicator, the UN's analysis summarizes the percentage of countries possessing measurements taken in two benchmark years: one year near the starting point of the MDGs (usually 1990) and another year nearer to the present (after 1999). Naturally, it is the difference of this pair of benchmark measurements, taken years apart, which proves whether progress is or is not being achieved for a particular MDG indicator.

The disappointing result of the UN's “Data Availability Analysis” is that, more often than not, the requisite pair of benchmark measurements doesn't exist, such that no factual conclusion about progress can be made. In the best case scenario, there are two indicators with paired benchmark measurements in 98% of countries. In the worst case scenario, there are 26 indicators—13 times as many—with paired benchmark measurements in none of the countries. Even the median MDG indicator, which is the one indicator most typical of the bunch, has paired benchmark measurements in just 5% of countries—meaning that the UN does not possess those data for 95% of countries.

In that context, the UN Millennium Project team is definitely wrong to believe that, health indicators aside, the MDGs “can be measured quite well” [1]. The UN's comprehensive analysis of all MDG indicators shows that, for whatever reason, most are not measured well, not even so rarely as twice a decade.

Why is measurement of the MDGs so generally poor? According to the UN Millennium Project team, the answer is money. They write that “developing countries and the international system,” which presumably includes the UN, “lack the resources to measure” the MDGs [1].

However, this belief, too, contradicts the evidence. Concerning the health MDGs, my article recommended expanding the network of demographic surveillance sites (DSS) as the single most efficient way to obtain timely, accurate measurements [2]. According to a recent study of DSS in Tanzania, this costs $0.01 per person, per year [4]. Thus, to institute DSS and good quality MDG measurements for the 4 billion poorest people worldwide would cost perhaps $40 million annually.

In this context, the UN Millennium Project team's argument that the “international system lacks the resources” to effectively measure the health MDGs is without credibility. The sum of $40 million is under 0.1% of the global foreign aid budget (Organization for Economic Cooperation and Development Development Assistance Committee [OECD DAC]).

Without such steps to measure progress on achieving the MDGs, any claims made for them are necessarily conjectural, rather than objective. The UN Millennium Project team writes that, even without measurement, “the MDGs are already promoting strengthened health systems in low-income countries” [1]—but regrettably, they fail to furnish evidence of this. They also write that “the very adoption of the maternal mortality goal … is provoking greatly increased attention to improvements in data collection” [1]. What they fail to mention is that the UN adopted that goal in 1990, and despite 15 years of provoking increased attention, elsewhere the UN Millennium Project team have called the data “unreliable” [5].

I could dispute other unsupported assertions in the reply of the UN Millennium Project team, but choose not to do so because it would distract from this fundamental point: whether to honor the health of the world's poorest or sickest people or to restore the earth's most vulnerable natural environments or to secure human rights for children and women, the UN must demonstrate much greater responsibility than it has in measuring the status of the MDGs. The UN Millennium Project team urges to “cement the MDGs as operational rather than simply rhetorical targets” [1]. I agree this is desirable, and, actually, to place rhetoric ahead of evidence is unethical.

That is why measurement to prove—not just to speculate—on the MDGs' operational progress cannot continue to be neglected, and also why the leading intellectuals of the UN Millennium Project err awfully in their judgment when justifying the neglect to date.
