# Correction: Author's Reply

**DOI:** 10.1371/journal.pmed.0030225

**Published:** 2006-05-30

**Authors:** Amir Attaran

In *PLoS Medicine*, volume 2, issue 11: DOI: 10.1371/journal.pmed.0020405

Reference 3 [United Nations (2005) Data availability analysis. New York: United Nations. Available: http://unstats.un.org/unsd/mi/techgroup/January2005/Series%20update%20status%20query_FC.xls. Accessed 13 October 2005] was publicly accessible at the time of publication of Amir Attaran's article, but is no longer publicly accessible. This file can now be found as Dataset S1.

Dataset S1 Millennium Indicators Database (41.5 KB XLS).

